# Replication of the 2023 radiologically isolated syndrome criteria in a multi-centre cohort

**DOI:** 10.1093/braincomms/fcaf323

**Published:** 2025-08-28

**Authors:** Friederike Held, Paula Uibel, Cornelius Berberich, Jonas Schaller-Nagengast, Vasil Mitrevski, Leo Hutter, Hayrettin Tumani, Joachim Havla, Christiane Gasperi, Mark Mühlau, Achim Berthele, Jan S Kirschke, Bernhard Hemmer

**Affiliations:** Technical University of Munich, School of Medicine and Health, Department of Neurology, TUM University Hospital, 81675 Munich, Germany; Technical University of Munich, School of Medicine and Health, Department of Neurology, TUM University Hospital, 81675 Munich, Germany; Technical University of Munich, School of Medicine and Health, Department of Neuroradiology, TUM University Hospital, 81675 Munich, Germany; Department of Neurology, University Hospital Ulm, 89081 Ulm, Germany; Ludwig-Maximilians-University, Institute of Clinical Neuroimmunology, LMU Hospital, 81377 Munich, Germany; Ludwig-Maximilians-University, Institute of Clinical Neuroimmunology, LMU Hospital, 81377 Munich, Germany; Department of Neurology, University Hospital Ulm, 89081 Ulm, Germany; Ludwig-Maximilians-University, Institute of Clinical Neuroimmunology, LMU Hospital, 81377 Munich, Germany; Technical University of Munich, School of Medicine and Health, Department of Neurology, TUM University Hospital, 81675 Munich, Germany; Technical University of Munich, School of Medicine and Health, Department of Neurology, TUM University Hospital, 81675 Munich, Germany; Technical University of Munich, School of Medicine and Health, Department of Neurology, TUM University Hospital, 81675 Munich, Germany; Technical University of Munich, School of Medicine and Health, Department of Neuroradiology, TUM University Hospital, 81675 Munich, Germany; Technical University of Munich, School of Medicine and Health, Department of Neurology, TUM University Hospital, 81675 Munich, Germany; Munich Cluster for Systems Neurology (SyNergy), 81377 Munich, Germany

**Keywords:** radiologically isolated syndrome, multiple sclerosis, risk factor, diagnostic criteria, conversion

## Abstract

Radiologically isolated syndrome (RIS) represents a pivotal stage for identifying individuals at high risk of transitioning into multiple sclerosis (MS). Early therapy initiation reduces the risk of conversion. The 2023-RIS criteria were proposed to identify presymptomatic individuals earlier. We aimed to replicate the diagnostic value of the 2023-RIS criteria in an independent cohort. In this retrospective cohort study, individuals diagnosed with RIS and longitudinally followed in three centres were stratified by the 2009- and 2023-RIS criteria. We conducted comparative analyses, including survival, hazard ratio and performance evaluations. Among *n* = 136 individuals, 27.2% converted to MS between 2009 and 2024 (observation time 55.4 months). We confirmed improved identification of individuals at risk using the 2023-RIS criteria (HR 4.30, *P* < 0.05; HR 4.71, *P* < 0.05) compared to 2009-RIS criteria (HR 1.32, *P* = 0.4; HR 1.43; *P* = 0.3) in 5- and 10-year intervals, respectively. 2023-RIS criteria demonstrated high sensitivity (94%) and negative predictive value (94%) but low specificity (29%). Adding CSF immunoglobulin G and M indices as an additional parameter following RIS diagnosis enhanced risk prediction specificity. We confirm the high sensitivity and predictive value of the 2023-RIS criteria for identifying individuals at risk of conversion to MS and suggest adding immunoglobulin indices to further improve specificity.

## Teaser

Held *et al*. report that the 2023 radiologically isolated syndrome criteria improve early identification of individuals at risk of multiple sclerosis conversion. In an independent cohort, adding cerebrospinal fluid immunoglobulin indices further increased risk prediction specificity, supporting their value in guiding individual treatment decisions.

## Graphical Abstract

**Graphical Abstract:**
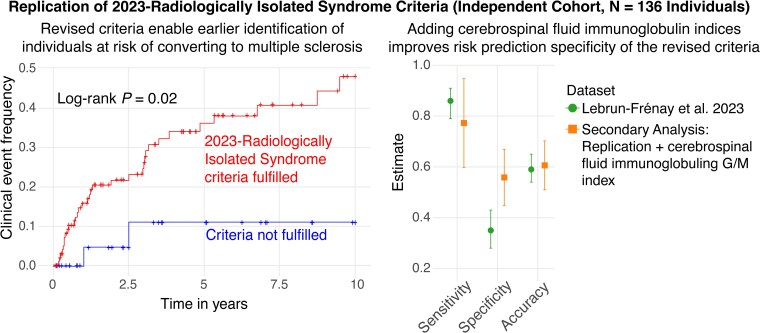

## Introduction

Radiologically isolated syndrome (RIS) refers to individuals with specific incidental demyelinating MRI lesions but no clinical symptoms, marking the earliest stage of identifying those at high risk for multiple sclerosis.^1^ Treatment trials within the last two years have shown beneficial effects of early treatment interventions in lowering the absolute risk of converting to multiple sclerosis.^2,3^ Individuals meeting the 2009-RIS criteria, which solely rely on MRI findings, have a 50% risk of converting to multiple sclerosis in an observation time of 10 years.^4,5^ Since reliable predictive biomarkers at the individual level are not yet available, parameters had to be defined for risk stratification.^6-8^ By incorporating established clinical and MRI parameters, the published 2023-RIS criteria, which were established in a large retrospective multi-centre cohort, proposed to better identify individuals at risk by allowing the diagnosis also to those with fewer MRI lesions if additional risk factors are met, specifically oligoclonal bands (OCBs), spinal cord lesions and evidence of dissemination in time (DIT) in any follow-up MRI.^9^ The revised 2023-RIS criteria showed high sensitivity but low specificity in identifying individuals who are at risk of developing multiple sclerosis.^9^

In this study, we aim to replicate the 2023-RIS criteria in an independent cohort. Additionally, we assessed supplementary parameters for further risk stratification following RIS diagnosis to guide individualized treatment regimes.

## Materials and methods

### Study criteria and cohort

A total of *n* = 136 individuals with suspected RIS based on their medical reports, diagnosed between 2009 and 2024 and identified from the databases of three multiple sclerosis centres [Ludwig-Maximillian University (LMU), University of Ulm (Ulm), Technical University of Munich (TUM)], were included in our analysis as they met the following criteria: at least one demyelinating-appearing lesion in a single specific location as defined by the 2017 McDonald criteria (periventricular, juxtacortical/cortical, infratentorial, or spinal) on the initial MRI (±6 months), available follow-up data and—if the 2009-RIS criteria were not fulfilled—information on at least two risk factors according to the 2023-RIS criteria to ensure reliable application of these criteria.^10^ Our inclusion strategy aligns with the selection criteria applied in the study by Lebrun-Frénay *et al*.,^9^ which included individuals with lesions in only one specific location but with additional specific features.

Individuals with suspected RIS based on their medical reports, meeting the above-mentioned inclusion criteria, but neither of the RIS definitions, had fewer MRI lesions to formally fulfil the 2009-RIS criteria and at least one, but not a combination, of risk factors required to fulfil the 2023-RIS criteria.

### CSF analysis

The levels of albumin, immunoglobulin (Ig) G (IgG) and M (IgM) in both CSF and serum were measured by nephelometry or turbidimetry (centre-dependent). Quantitative assessment of the intrathecal humoral immune response was performed by calculating the Ig-index for IgG and IgM using the formula Ig_x_ Index = QIg_x_/Qalb with QIg_x_ for either IgG or IgM and Qalb as the CSF-to serum Ig and albumin quotient, respectively. An IgG index > 0.70 and an IgM index > 0.10 were considered elevated. Detection of OCBs was performed using isoelectric focusing followed by immunoblotting, immunofixation, or, rarely, silver staining (centre-dependent). OCBs were considered positive if patterns 2 or 3 were present.^11^

### Brain and spinal cord MRI assessment

To ensure consistency and comparability of MRI findings, all images were analysed by two blinded independent neuroradiologists at TUM.

Images included were selected hierarchically: if available, cerebral MRI scans acquired with 3 T, 1–3 mm slice thickness and T2-weighted FLAIR sequences were prioritized. If unavailable, 1.5 T MRI scans with 5 mm slice thickness and T1-native sequences were used. Additionally, spinal MRI scans were analysed, when available, using sagittal and axial T2-weighted imaging sequences with a slice thickness of 3 mm. Contrast-enhanced sequences were included in the MRI assessment when available.

Lesions were documented by region (periventricular, juxtacortical, infratentorial, spinal) numerically on a scale from zero to eight or categorical as ‘≥9’ if exceeded. Lesion patterns (vascular, inflammatory, mixed) and gadolinium-enhancing lesions (GAD+) were documented separately.

### Statistical analysis

We stratified the cohort into three groups based on (i) fulfilling the 2009-RIS criteria (2009-RIS); (ii) not fulfilling the 2009-RIS but meeting 2023-RIS criteria (2023-RISonly); and (iii) meeting neither (Pre-RIS). The first two groups (2009-RIS, 2023-RISonly) together represent the cohort fulfilling the revised 2023-RIS criteria (2023-RIS) (Supplementary Fig. 1). Statistical analyses were conducted using R Version 4.3.2. The alpha risk was set to 0.05 and adjusted for multiple testing following *post hoc* comparison (Bonferroni).

For group comparisons, chi-square/Fisher’s exact tests were used for categorical and Wilcoxon rank-sum/Kruskal–Wallis test for numerical variables (non-normally distributed data by Shapiro–Wilk), with Tukey’s HSD/Dunn’s test *post hoc* analyses as needed. A logistic regression model assessed predictors of multiple sclerosis conversion, while univariate Cox proportional hazard models evaluated additional risk factors for early conversion.

For exploratory analysis of CSF indices as continuous variables, we applied a refined tertile stratification of index values based on their distribution using the ‘stats’ R package. To increase resolution, we further subdivided the upper tertile at its median value, resulting in four categories: low, mid, high-lower and high-upper.

Kaplan–Meier curves assessed time to multiple sclerosis conversion and cumulative probability at 5- and 10-year intervals, treating non-converters with adequate follow-up as controls. Cox proportional hazards and log-rank tests assessed group differences, including differences among the CSF index stratified groups.

The 2023-RIS criteria’s performance was evaluated via sensitivity, specificity, predictive values, overall accuracy and area under the curve (AUC) for 5- and 10-year periods. A secondary analysis assessed additive risk factors after RIS diagnosis.

### Standard protocol approvals, registrations and patient consent

This study received approval from the ethics commission of the TUM (466/15), LMU (24-0891) and Ulm (20/10) and followed the principles outlined in the Declaration of Helsinki. All patients provided written informed consent. The research was conducted and reported following the STROBE guidelines for observational studies.

## Results

### Distinct features of 2009-RIS and 2023-RISonly cohorts

Out of *n* = 136 individuals, *n* = 105 (77.2%) fulfilled the 2023-RIS criteria (Table 1). Among them, *n* = 76 individuals met the 2009-RIS criteria, and *n* = 29 did not fulfil the 2009-RIS criteria but the 2023-RIS criteria (2023-RISonly) (Supplementary Fig. 2). Individuals of the 2023-RISonly cohort tended to be younger (age < 37 years: 75.9% versus 52.6%) with a longer time to multiple sclerosis conversion (mean [SD]: 37.8 months [33.0] versus 29.1 [39.6]) compared to 2009-RIS, but this difference was not statistically significant. Fulfilment of the dissemination in time criteria, whether in CSF (OCB 100% versus 76.1%, *P* < 0.01) or MRI (new T2-lesion 96.4% versus 67.6%, *P* < 0.01), was more frequent in the 2023-RISonly cohort, except for contrast-enhancing MRI lesions. Contrast-enhancing lesions at initial MRI, along with spinal cord lesions, were more prevalent in the 2009-RIS cohort. No differences were observed regarding the reason for the initial MRI, family history of multiple sclerosis, or disease-modifying treatment (DMT) before multiple sclerosis conversion. While there was a trend towards longer conversion times in the 2023-RISonly group, no statistically significant differences were found in the time interval to multiple sclerosis conversion among the three groups.

**Table 1:** Demographic, clinical and MRI characteristics of the RIS cohort

Variables	Total (*n* = 136)	2009-RIS (*n* = 76)^a^	2023-RISonly (*n* = 29)^a^	Pre-RIS (*n* = 31)	2009-RIS versus 2023-RISonly^b^	2023-RIS versus Pre-RIS^c^
Age < 37 years (%)	81 (59.6%)	40 (52.6%)	22 (75.9%)	19 (61.3%)	*P* = 0.16	*P* = 0.99
Female sex (%)	83 (61.0%)	48 (63.2%)	17 (58.6%)	18 (58.1%)	*P* = 1.00	*P* = 0.86
Family history positive (%)	16 (12.0%) (3NA)	11 (14.9%)	3 (10.3%)	2 (6.7%)	*P* = 1.00	*P* = 0.52
Reason MRI available^d^ (%)	136 (100%)	76 (100%)	29 (100%)	31 (100%)	*P* = 1.00	*P* = 0.73
CSF IgM index positive (%)	25 (27.8%) (46NA)	14 (29.8%)	7 (38.9%)	4 (16.0%)	*P* = 1.00	*P* = 0.19
CSF IgG index positive (%)	45 (45.5%) (37NA)	29 (51.8%)	10 (55.6%)	6 (24.0%)	*P* = 0.99	***P*** **<** **0.05**
CSF OCB positive (%)	90 (68.7%) (5NA)	54 (76.1%)	29 (100%)	7 (22.1%)	***P*** **<** **0.01**	***P*** **<** **0.01**
≥1 GAD+ lesion MRI^d^ (%)	26 (21.3%) (14NA)	24 (35.8%)	2 (7.4%)	0 (0%)	***P*** **<** **0.05**	***P*** **<** **0.01**
New T2-lesion fu-MRI^e^ (%)	79 (63.2%) (11NA)	46 (67.6%)	27 (96.4%)	6 (20.7%)	***P*** **<** **0.01**	***P*** **<** **0.01**
GAD+ lesion fu-MRI^e^ (%)	15 (12.1%) (12NA)	10 (14.7%)	5 (18.5%)	0 (0%)	*P* *=* 1.00	***P*** ***<*** **0.05**
≥1 sMRI lesion^d^ (%)	46 (46.0%) (36NA)	36 (64.3%)	9 (47.4%)	1 (4.0%)	*P* = 0.91	***P*** **<** **0.01**
DMT before multiple sclerosis (%)	30 (22.1%)	20 (26.3%)	8 (27.6%)	2 (6.5%)	*P* = 1.00	***P*** **<** **0.05**
Conversion to multiple sclerosis (%)	37 (27.2%)	22 (28.9%)	13 (44.8%)	2 (6.5%)	*P* = 0.57	***P*** **<** **0.01**
Time to multiple sclerosis (mo) mean [SD]	31.7 [36.1]	29.1 [39.6]	37.8 [33.0]	21.0 [12.7]	*P* = 0.37	*P* = 1.00
Obs. period (mo) mean [SD]	55.4 [49.5]	53.3 [51.0]	73.6 [49.9]	43.6 [41.2]	*P* = 0.07	*P* = 0.20

Results reflect available data, as some variables contained missing values. Categorical variables were compared using chi-square test or Fisher’s exact test. Numeric variables were non-normally distributed (Shapiro–Wilk) and compared using Wilcoxon rank-sum (two groups) or Kruskal–Wallis (three groups). Bold values indicate statistical significance (*P* < 0.05).

DMT, disease-modifying treatment; fu, follow-up; GAD, gadolinium; IgG, immunoglobulin G; IgM, immunoglobulin M; mo, month; NA, not available; Obs., observation; OCB, oligoclonal bands; SD, standard deviation; sMRI, spinal cord MRI.

^a^Part of the revised 2023-RIS criteria.

^b^Statistical significance displayed for 2009-RIS versus 2023-RISonly arose from three subgroup comparisons with *post hoc* pairwise comparisons. Level of significance was set to 0.05 (Bonferroni).

^c^Statistical significance displayed following two group comparisons. Level of significance was set to 0.05.

^d^Refers to the initial MRI scan.

^e^Refers to any follow-up MRI performed before conversion to multiple sclerosis.

Individuals not meeting either RIS criteria rarely had CSF OCB (22.1%) and no active MRI lesions. Despite not fulfilling either set of criteria, two individuals converted to multiple sclerosis, highlighting the concept of ‘Pre-RIS’—a term used to describe individuals with fewer MRI abnormalities or risk factors than required to formally meet the 2009- or 2023-RIS criteria, but who are considered at elevated risk of multiple sclerosis and warrant clinical monitoring.^12^ Both individuals had complete data for all risk factors defined by the 2023-RIS criteria.

### Dissemination in time independently predicts conversion to multiple sclerosis

The novelty of the 2023-RIS criteria is the implementation of the CSF parameter in addition to MRI findings and taking follow-up MRIs into account. Consistent with the original study, we found that CSF OCBs (OR 6.99, [CI: 2.00–24.45]) and DIT in any follow-up MRI (new T2-lesions: OR 4.50, [CI: 1.60–12.69], GAD+: OR 7.47, [CI: 2.33–24.05]) are independent risk factors of conversion to multiple sclerosis (*P*< 0.01) (Supplementary Table 1). In univariate analysis, the CSF IgG index (OR 4.08, [CI: 1.50–11.05], *P* < 0.05) was associated with conversion to multiple sclerosis at any time but was not independent of the risk factors CSF OCBs or DIT on follow-up MRI. The odds of conversion tended to be higher in the presence of GAD+ lesions (OR 1.68, [CI: 0.66–4.28], *P* = 0.28) and spinal cord lesions (OR 1.71, [CI: 0.69–4.26], *P* = 0.25) on the initial MRI. Furthermore, younger individuals demonstrated a trend towards increased likelihood of conversion to multiple sclerosis (OR 1.89, [CI: 0.84–4.25], *P* = 0.12).

### Improved discrimination of individuals at risk of conversion to multiple sclerosis

**Figure 1:**
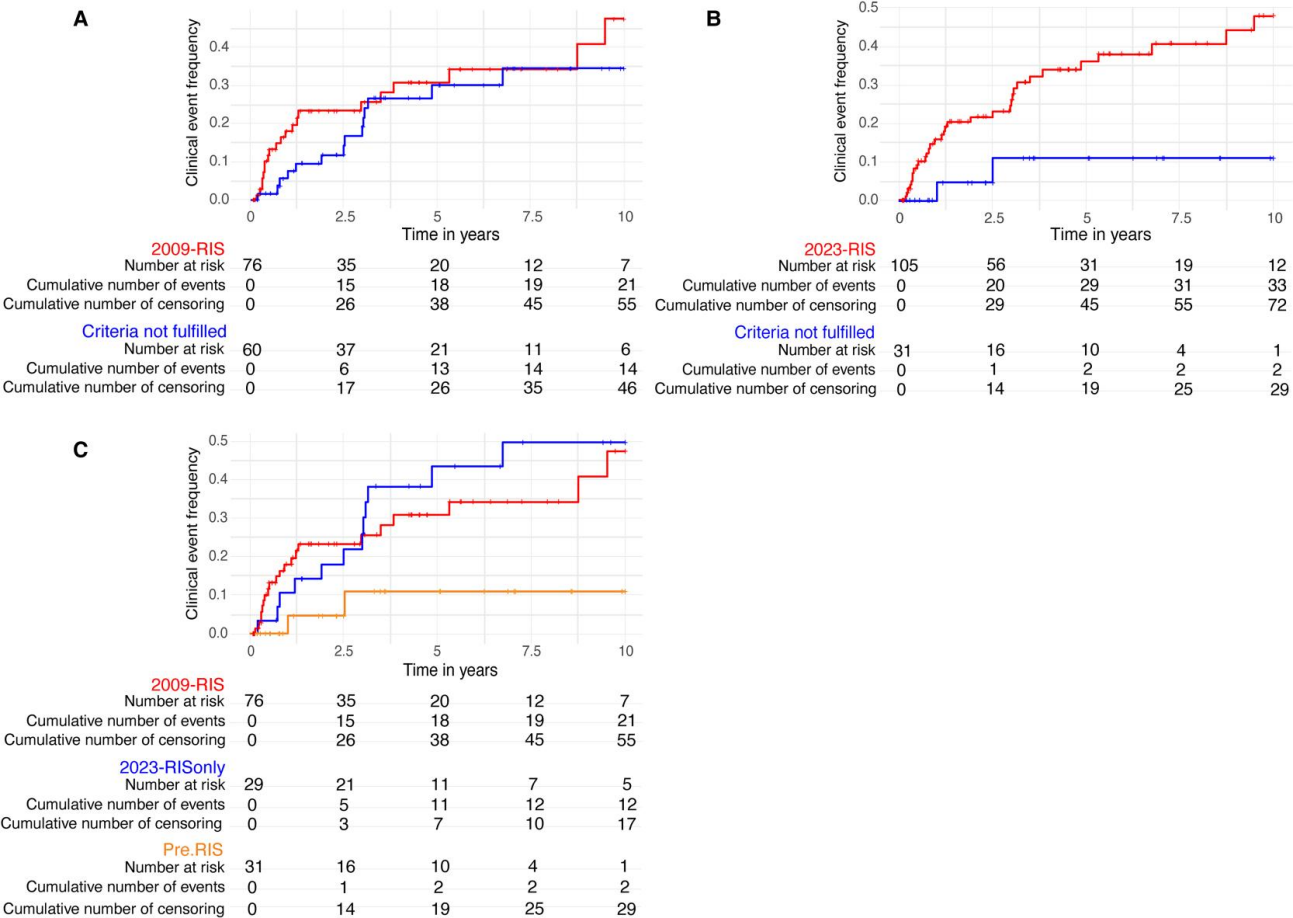
**Kaplan–Meier curves illustrating cumulative event frequencies in a 10-year interval stratified by subgroups.** (**A**) Individuals meeting the 2009-RIS criteria (upper curve in red, *n* = 76) had a marginally higher risk of conversion to multiple sclerosis compared to those not fulfilling the criteria (lower curve in blue, *n* = 60) (HR 1.43 [95% CI: 0.73–2.82], log-rank *P* = 0.3). (**B**) The 2023-RIS criteria (upper curve in red, *n* = 105) demonstrated significantly enhanced discriminatory power, with individuals meeting the criteria showing over a 4-fold increased risk of conversion to multiple sclerosis (HR 4.71 [95% CI: 1.13–19.65], log-rank *P*  *<* 0.05) compared to those not fulfilling the criteria (lower curve in blue, *n* = 31). (**C**) In a three-group comparison, both 2009-RIS (intermediate curve at 10 year in red, *n* = 76) (HR 4.55 [95% CI: 1.07–19.44], log-rank *P*  *<* 0.05) and 2023-RISonly criteria (upper curve at 10 year in blue, *n* = 29) (HR 5.01 [95% CI: 1.12–22.43], log-rank *P*  *<* 0.05) were associated with a substantial higher 10-year multiple sclerosis risk compared to individuals not meeting any criteria (Pre-RIS) (lower curve in orange, *n* = 31).

When we applied the 2009-RIS criteria to the entire cohort, a substantial proportion of individuals (*n* = 14) not fulfilling the criteria converted to multiple sclerosis in an observation time of 10 years. Accordingly, individuals fulfilling these criteria had only a 1.4-fold higher risk (HR 1.43 [CI: 0.73–2.82], log-rank *P* = 0.3) of converting to multiple sclerosis compared to the non-2009-RIS cohort (Fig. 1A). In contrast, the 2023-RIS criteria (2009-RIS and 2023-RISonly) demonstrated an improved ability to identify individuals at risk over a 10-year observation period (Fig. 1B). Individuals meeting the 2023-RIS criteria were more than four times as likely to convert to multiple sclerosis (HR 4.71; [CI: 1.13–19.65], log-rank *P* < 0.05) compared to those not fulfilling the revised criteria. The discrimination between groups remained consistent over time, meeting the Schoenfeld residual assumptions. Only two individuals in the cohort who did not meet the 2023-RIS criteria converted to multiple sclerosis.

In a three-group comparison (2009-RIS, 2023-RISonly, Pre-RIS), both RIS criteria independently demonstrated a significantly higher 10-year risk for conversion to multiple sclerosis compared to Pre-RIS individuals (2009-RIS: HR 4.55 [CI: 1.07–19.44], log-rank *P* < 0.05; 2023-RISonly: HR 5.01 [CI: 1.19–22.43], log-rank *P* < 0.05) (Fig. 1C). There was no substantial difference between the two criteria when directly compared. The clinical event frequency within 2.5 years following RIS diagnosis was highest in the 2009-RIS cohort. In contrast, the clinical event frequency beyond 2.5 years was higher in the subgroup fulfilling the 2023-RISonly criteria.

### Additional CSF parameters enhance specificity of RIS criteria

The cumulative incidence of conversion to multiple sclerosis at 10 years was comparable when applying the 2009-RIS (47% [CI: 23.7–63.7]) or 2023-RIS (48% [CI: 31.7–60.3]) criteria. However, differences became evident in performance analysis. The 2023-RIS criteria demonstrated higher sensitivity (94.3%) for detecting individuals at risk in a 10-year interval. Additionally, the negative predictive value was remarkably high (93.5%), effectively ruling out individuals truly not at risk (Table 2, Supplementary Table 2). However, the criteria showed low specificity (28.7%), highlighting the need for further refinement with additional parameters.

**Table 2:** Performance of 2023-RIS criteria at 5- and 10-year intervals

Parameter (% [95% CI])	Revised criteria^a^	Replication	Replication
	5-Year	5-Year	10-Year
Sensitivity	86.0% [79.4; 91.1]	93.5% [84.9; 100.0]	94.3% [86.6; 100.0]
Specificity	35.4% [28.0; 43.3]	27.6% [19.1; 36.2]	28.7% [19.9; 37.5]
Positive predictive value	55.4% [52.1; 58.6]	27.6% [19.1; 36.2]	31.4% [22.5; 40.3]
Negative predictive value	73.1% [63.4; 80.9]	93.5% [84.9; 100.0]	93.5% [84.9; 100.0]
Accuracy	59.8% [54.1; 65.3]	42.6% [34.3; 51.0]	45.6% [37.2; 54.0]
Area under the curve	60.7% [55.0; 66.2]	60.6% [54.4; 60.6]	61.5% [55.6; 61.5]

^a^Lebrun-Frénay *et al*.^9^

**Figure 2:**
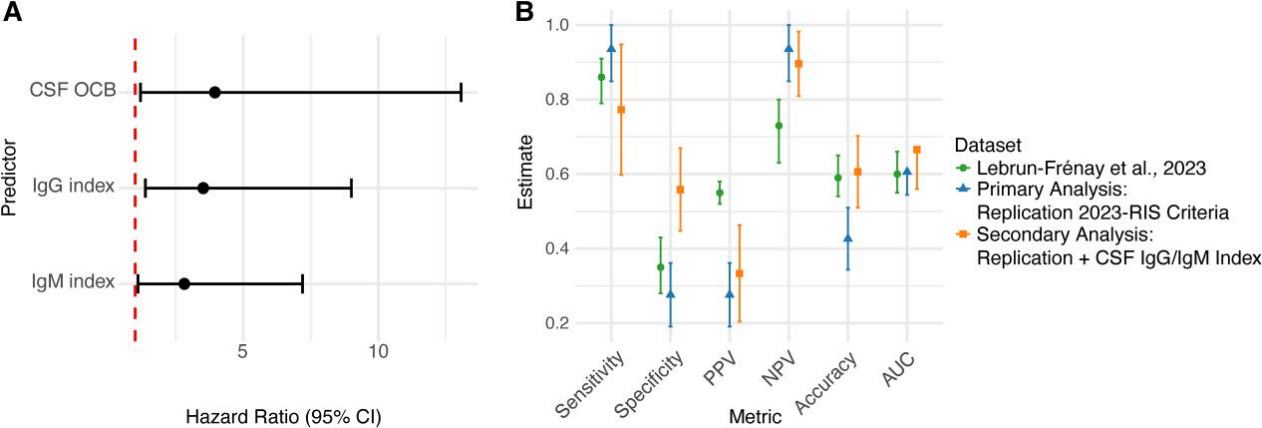
**Predictive ability of CSF indices for early conversion to multiple sclerosis in a 5-year interval.** (**A**) Hazard ratios (HR) with 95% confidence intervals (CI) as estimated by univariate Cox proportional hazards models for OCB, IgG index and IgM index in predicting multiple sclerosis conversion in a five-year interval. The red dashed line indicates an HR of one. (**B**) Performance metrics for different datasets: published dataset by Lebrun-Frénay *et al*.^9^ (left bar in green), dataset of the primary analysis of our replication cohort (middle bar in blue) (*n* = 136) and secondary analysis of our replication cohort with incorporation of CSF IgM/IgG indices (right bar in orange) (*n* = 99). In the orange cohort, individuals meeting the 2023-RIS criteria in the primary analysis and having a positive IgG or IgM index were classified as RIS to assess the additive value of CSF indices in enhancing specificity. CSF, cerebrospinal fluid; IgG, immunoglobulin G; IgM, immunoglobulin M; OCBs, oligoclonal bands; PPV, positive predictive value; NPV, negative predictive value; AUC, area under the curve.

Given the association of a positive IgG index with conversion to multiple sclerosis at any time, with a similar trend also shown for the IgM index, we assessed their additive value in enhancing specificity and predicting early multiple sclerosis conversion. CSF OCB and both Ig indices were associated with conversion to multiple sclerosis at 5 years in univariate analysis (OCB: HR 4.0 [CI: 1.19–13.06], *P* < 0.05; IgG index: HR 3.5 [CI: 1.38–9.00], *P* < 0.01; IgM index: HR 2.8 [CI: 1.11–7.19], *P* < 0.05) (Fig. 2A). Refining risk stratification and stratifying the cohort by adding a positive IgG or IgM index to the revised criteria lowered sensitivity (77.3%) but increased specificity (55.8%) in a 5-year interval (Fig. 2B, Supplementary Table 3). Therefore, the CSF IgG or IgM index might improve risk stratification following RIS diagnosis. As an exploratory analysis, we also modelled the IgG index as a continuous variable, which was associated with multiple sclerosis conversion risk at both 5- and 10-year intervals in univariate Cox regression (HR 4.59 [CI: 1.41–14.94], *P* < 0.01; HR 4.75 [CI: 1.48–15.28], *P* < 0.01, respectively), and revealed a trend suggestive of a dose-dependent effect within tertile-stratified subgroups with respect to multiple sclerosis conversion risk (Supplementary Fig. 3).

## Discussion

In this retrospective cohort study, we replicated the 2023-RIS criteria in a multi-centre cohort, demonstrating high sensitivity, strong negative predictive value and improved discrimination of individuals at risk of conversion to multiple sclerosis compared to the 2009-RIS criteria. However, the specificity remained low. To enhance specificity, CSF IgG and IgM indices were identified as suitable parameters for further risk stratification following RIS diagnosis.

We confirmed OCBs and DIT in any follow-up MRI as independent risk factors of conversion to multiple sclerosis. Both parameters are known risk factors for multiple sclerosis and are part of the current multiple sclerosis diagnosis criteria.^10,13,14^ Additionally, younger age at RIS diagnosis, the presence of spinal cord lesions and GAD+ lesions on initial MRI tend to be associated with an increased risk of multiple sclerosis in our analysis. The connection between younger age and risk of MS conversion is well established, whereas the associations with spinal cord involvement and GAD+ lesions at baseline are inherently reported.^4,15-18^

Individuals fulfilling the 2009-RIS criteria tended to be older, and a higher proportion converted shortly after RIS diagnosis compared to the 2023-RIS criteria. This supports findings from the original study, where the revised criteria capture individuals with RIS at an earlier stage.^9^ Earlier detection opens the possibility for early treatment interventions, as recent clinical trials have shown beneficial effects in profoundly lowering the risk of conversion to multiple sclerosis.^2,3^ However, to guide treatment decisions effectively, greater specificity or further stratification following RIS diagnosis is necessary to identify high-risk individuals and maintain a positive benefit-risk balance.

The 2023-RIS criteria demonstrated exceptionally high sensitivity and negative predictive value but low specificity at both 5- and 10-year intervals, consistent with the original study. The low specificity likely reflects the broader and more heterogeneous at-risk population defined by the revised 2023-RIS criteria, which increases the overall number of cases and shifts the case-to-control ratio, thereby reducing specificity. The higher sensitivity observed in our dataset as compared to the original study may be attributed to several factors: (i) the highly controlled cohort, involving three centres in one country compared to 37 globally in the original study; (ii) using OCB alone as a risk factor, rather than OCB or IgG index; and (iii) the completeness of OCB data in our cohort (96.3% versus 54.6%). OCBs are known to be more sensitive but less specific than quantitative IgG assessments for predicting conversion to clinically defined multiple sclerosis, and relying solely on OCB likely contributed to the high sensitivity observed in our analysis.^19,20^

Building on the inverse relationship between sensitivity and specificity, we evaluated quantitative IgG and IgM indices to improve specificity. A positive CSF IgG index was associated with conversion to multiple sclerosis at any time, while both indices were associated with early conversion within 5 years.

Previous studies have demonstrated that a CSF IgG index > 0.7 increases the likelihood of conversion to multiple sclerosis in individuals with clinically isolated syndrome.^21,22^ While quantitative IgG assessments have lower sensitivity than OCB, their specificity is notably higher in predicting conversion to multiple sclerosis.^19,22^ Incorporating CSF IgG and IgM indices increased the specificity of the criteria to 55.8% at 5 years, although sensitivity decreased to 77.3%. While these findings suggest that CSF indices may not be ideal for inclusion in diagnostic criteria aimed at detecting all individuals at risk, they are valuable for refining risk stratification following an RIS diagnosis. Of note, we observed a dose-dependent trend between higher IgG index values and increased multiple sclerosis conversion risk, similar to findings reported for the kappa-free light chain (κ-FLC) index.^23,24^

We confirmed the robustness of the 2023-RIS criteria and a comparable 10-year cumulative risk to the 2009-RIS criteria, aligning with prior findings.^4^ However, our study has limitations with respect to cohort size and completeness of data for some variables. The low prevalence of RIS and the absence of standardized diagnostic workflows due to the retrospective study design are likely reasons for that. Nonetheless, strict inclusion criteria ensured that missing values in the pre-RIS group did not affect the performance evaluation of the 2023-RIS criteria in our cohort. Low accuracy and moderate AUC, the latter similar to published criteria, reflect challenges in applying the diagnostic criteria to our imbalanced dataset, where converters were substantially outnumbered by non-converters, with many censor events. Additionally, the MRI data varied widely, with heterogeneous and limited sequences for a substantial portion of the cohort. This made it moreover impossible to reliably apply the newly proposed McDonald criteria to our cohort, which suggests defining RIS as multiple sclerosis in specific situations.

Diagnostic guidelines are necessary for comprehensive and consistent prospective data collection to address these limitations in future trials and uniformly identify and monitor individuals with RIS. Improving the comparability and clinical relevance of RIS criteria will also depend on standardized MRI protocols and CSF diagnostic procedures. Automated imaging tools could ensure consistent lesion burden assessments and incorporation of additional risk parameters.^25,26^ The κ-FLC index, recently shown to be associated with multiple sclerosis symptoms when exceeding 6.1, is a promising substitute for OCB due to its quantitative nature and observer independence.^27-29^ Establishing a risk pyramid incorporating genetic and environmental factors may help prioritize treatment for high-risk individuals with RIS.

In conclusion, this study replicated the 2023-RIS criteria, confirming their high sensitivity and strong negative predictive value. To address the low specificity, we propose incorporating CSF indices for further risk stratification following RIS diagnosis to guide treatment decisions.

## Supplementary Material

fcaf323_Supplementary_Data

## Data Availability

Anonymized data not published within this article will be made available by reasoned request in the context of data transfer agreements. The code generated for this work is available at https://osf.io/6xqdc/?view_only=1410578d90884d63a99108b3776534b7.
