# Correction to: Replication of the 2023 radiologically isolated syndrome criteria in a multi-centre cohort

**DOI:** 10.1093/braincomms/fcaf392

**Published:** 2025-10-30

This is a **correction** to:

Friederike Held, Paula Uibel, Cornelius Berberich, Jonas Schaller-Nagengast, Vasil Mitrevski, Leo Hutter, Hayrettin Tumani, Joachim Havla, Christiane Gasperi, Mark Mühlau, Achim Berthele, Jan S Kirschke, Bernhard Hemmer, Replication of the 2023 radiologically isolated syndrome criteria in a multi-centre cohort, *Brain Communications*, Volume 7, Issue 5, 2025, fcaf323, https://doi.org/10.1093/braincomms/fcaf323

There was a minor methodological adjustment to Table 1.

While this manuscript states that the Bonferroni adjustment was applied for three-group comparisons, the categorical variables used Benjamini-Hochberg adjustment, whereas numerical variables correctly used Bonferroni as stated.

This does not affect any conclusions - all significance patterns remain identical between both methods and for methodological consistency, table 1 has been updated with consistent Bonferroni adjustment throughout.

