# Supplementary material for: Replication of the 2023 radiologically isolated syndrome criteria in a multi-centre cohort
Source: Brain Commun. 2025 Aug 28;7(5):fcaf323. doi: 10.1093/braincomms/fcaf323 (PMC12417997; doi:10.1093/braincomms/fcaf323)
Supplement: fcaf323_Supplementary_Data [file fcaf323_supplementary_data.docx]

**Supplementary Material**


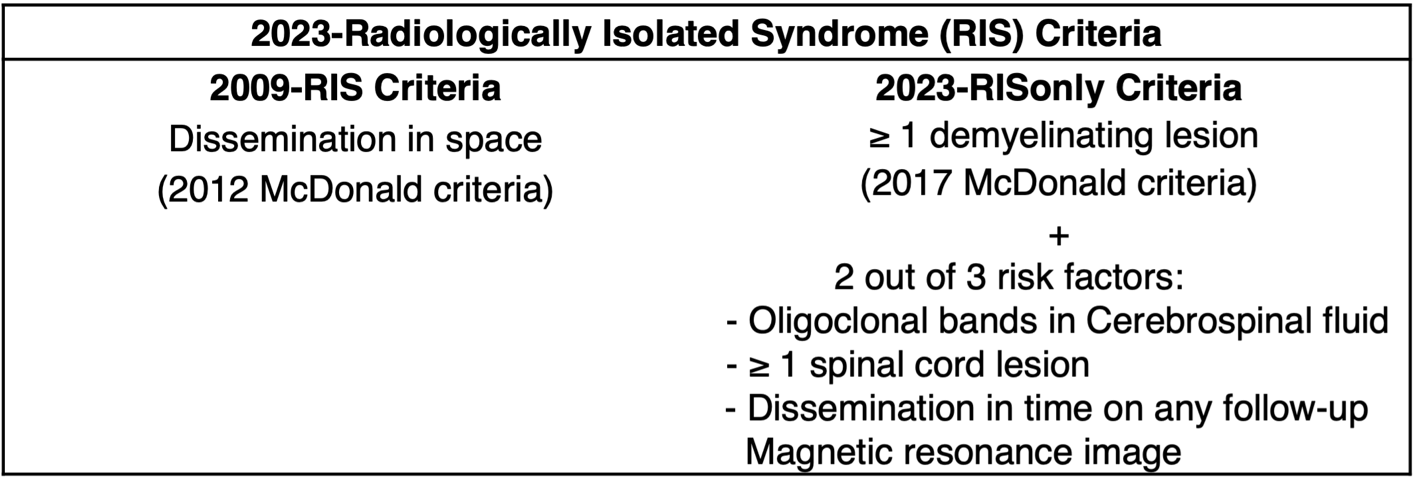


**Supplementary Figure 1: Overview of the 2023-RIS Criteria published by Lebrun-Frénay et al.^1^**


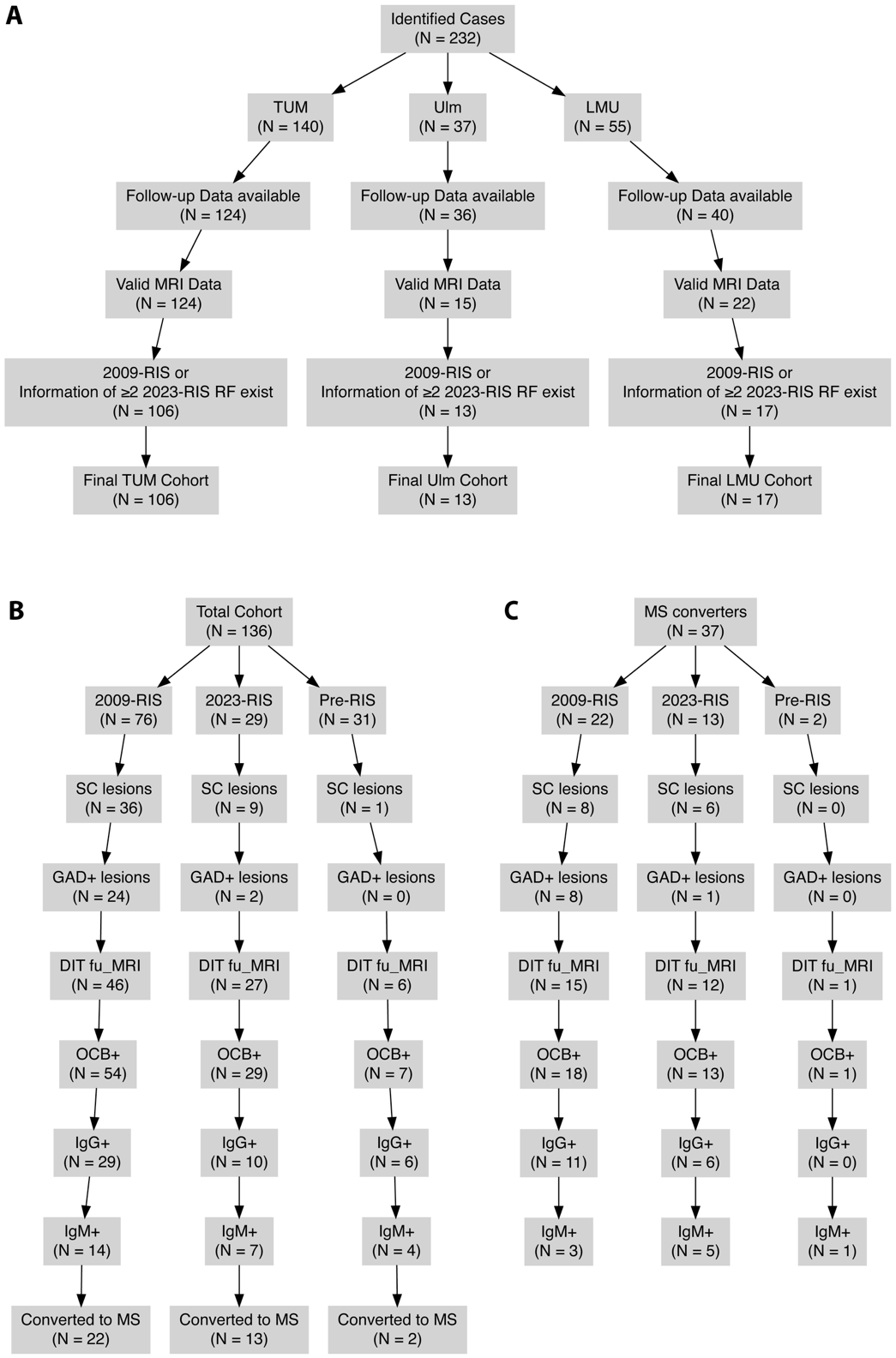


**Supplementary Figure 2: Flowchart illustrating cohort selection, final cohort composition, and characteristics of subgroups.** (A) Flowchart showing patient inclusion at three study centers (TUM, Ulm, LMU), with stepwise exclusions based on availability of follow-up data, valid MRI scans, and fulfillment of either the 2009-RIS criteria or available information of ≥2 risk factors as defined by the 2023-RIS criteria. (B) Risk profiles of the full study cohort (N = 136), stratified by subgroups (2009-RIS, 2023-RIS, and Pre-RIS), showing selected clinical and imaging features as well as the number of individuals who converted to MS. (C) The same stratification and parameters were displayed for the subgroup of MS converters only (N = 37). **Abbreviations:** DIT – dissemination in time; fu_MRI – follow-up magnetic resonance imaging; GAD+ - gadolinium-enhancing; IgG+ / IgM+ - positive CSF indices of IgG or IgM; MS - Multiple Sclerosis; OCB+ - positive oligoclonal bands; RIS - Radiologically isolated syndrome; RF - risk factors; SC = spinal cord.

**Supplementary Table 1: Risk factors of conversion to MS in univariate and multivariate logistic regression analysis**

| **Risk Factor** | **Univariate OR [95%CI]** | **Univariate**  ***P^a^*** | **Multivariate**  **OR [95%CI] (Model1*)** | **Multivariate *P^a^***  **(Model1*)** | **Multivariate OR [95%CI] (Model2**)** | **Multivariate *P^a^* (Model2**)** |
| --- | --- | --- | --- | --- | --- | --- |
| CSF OCB positive | 6.99 [2.00; 24.45] | **<0.01** | 9.74 [1.14; 83.12] | **<0.05** |  |  |
| New T2-lesion fu-MRI^b^ | 4.50 [1.60; 12.69] | **<0.01** | 7.63 [1.55; 37.49] | **<0.05** | 3.26 [0.60; 17.59] | 0.17 |
| GAD+ lesion fu-MRI^b^ | 7.47 [2.33; 24.05] | **<0.01** |  |  |  |  |
| CSF IgG index positive | 4.08 [1.50; 11.05] | **<0.05** |  |  | 4.01 [0.92; 17.45] | 0.06 |
| CSF IgM index positive | 2.76 [0.97; 7.83] | 0.06 |  |  |  |  |
| Age <37 years | 1.89 [0.84; 4.25] | 0.12 |  |  |  |  |
| ≥1 sMRI lesion^c^ | 1.71 [0.69; 4.26] | 0.25 | 0.71 [0.23-2.21] | 0.55 | 1.98 [0.48; 8.15] | 0.34 |
| ≥1 GAD+ lesion MRI^c^ | 1.68 [0.66; 4.28] | 0.28 |  |  |  |  |
| DMT before MS | 1.46 [0.61; 3.51] | 0.39 |  |  |  |  |
| Family history positive | 1.21 [0.39; 3.75] | 0.74 |  |  |  |  |
| Female Sex | 0.91 [0.42; 1.98] | 0.82 |  |  |  |  |

^a^ The level of significance was set to 0.05

^b^ Refers to any follow-up MRI performed before conversion to MS.

^c^ Refers to the initial MRI scan.

* Multivariate analysis with the independent predictors defined in the study by Lebrun-Frénay C *et al.^1^:* OCB + new T2-lesions in follow-up MRI + ≥1 spinal cord lesion

** Multivariate analysis with the predictors: CSF IgG index positive + new T2-lesions in follow-up MRI *or* GAD+ lesion fu-MRI + ≥1 spinal cord lesion

Results reflect available data, as some variables contained missing values.

CI – Confidence Interval, DMT – Disease-modifying treatment, fu – follow-up, GAD - Gadolinium, IgG – Immunoglobulin G, IgM – Immunoglobulin M, OCB – oligoclonal bands, OR – Odds Ratio, sMRI – spinal cord MRI

**Supplementary Table 2: Performance of 2009-RIS, 2023-RISonly and revised 2023-RIS criteria at 10-year intervals.**

| **Parameter** (% [95%CI]) | **2009-RIS versus not** | **2023-RISonly versus not** | **2023-RIS versus not** |
| --- | --- | --- | --- |
| **Sensitivity** | 60.0% [43.8; 76.2] | 34.3% [18.6; 50.0] | 94.3% [86.6; 100.0] |
| **Specificity** | 45.5% [35.8; 55.3] | 83.2% [75.9; 90.5] | 28.7% [19.9; 37.5] |
| **Positive predictive value** | 27.6% [17.6; 37.7] | 41.4% [23.5; 59.3] | 31.4% [22.5; 40.3] |
| **Negative predictive value** | 76.7% [66.0; 87.4] | 78.5% [70.7; 86.3] | 93.5% [84.9; 100.0] |
| **Accuracy** | 49.3% [40.9; 57.7] | 70.6% [62.9; 78.2] | 45.6% [37.2; 54.0] |
| **Area under the curve** | 52.8% [43.2; 52.8] | 58.7% [49.9; 58.7] | 61.5% [55.6; 61.5] |
| **Case:Control ratio** | 76 vs 60 | 29 vs 107 | 105 vs 31 |

**Supplementary Table 3: Performance of 2023-RIS criteria with primary and secondary analyses at a 5-year interval**

| **Parameter** (% [95%CI]) | **Revised Criteria^a^**  **(*n* = 288)** | **Replication**  **(*n* = 136)** | **Secondary analysis**  **Replication**  **(+CSF Ig Index)**  **(*n* = 99)** |
| --- | --- | --- | --- |
| Sensitivity | 86.0% [79.4; 91.1] | 93.5% [84.9; 100.0] | 77.3% [59.8; 94.8] |
| Specificity | 35.4% [28.0; 43.3] | 27.6% [19.1; 36.2] | 55.8% [44.8; 66.9] |
| Positive predictive value | 55.4% [52.1; 58.6] | 27.6% [19.1; 36.2] | 33.3% [20.4; 46.3] |
| Negative predictive value | 73.1% [63.4; 80.9] | 93.5% [84.9; 100.0] | 89.6% [80.9; 98.2] |
| Accuracy | 59.8% [54.1; 65.3] | 42.6% [34.3; 51.0] | 60.6% [51.0; 70.2] |
| Area under the Curve | 60.7% [55.0; 66.2] | 60.6% [54.4; 60.6] | 66.6% [56.0; 66.6] |

^a^ Lebrun-Frénay *et al*.^1^


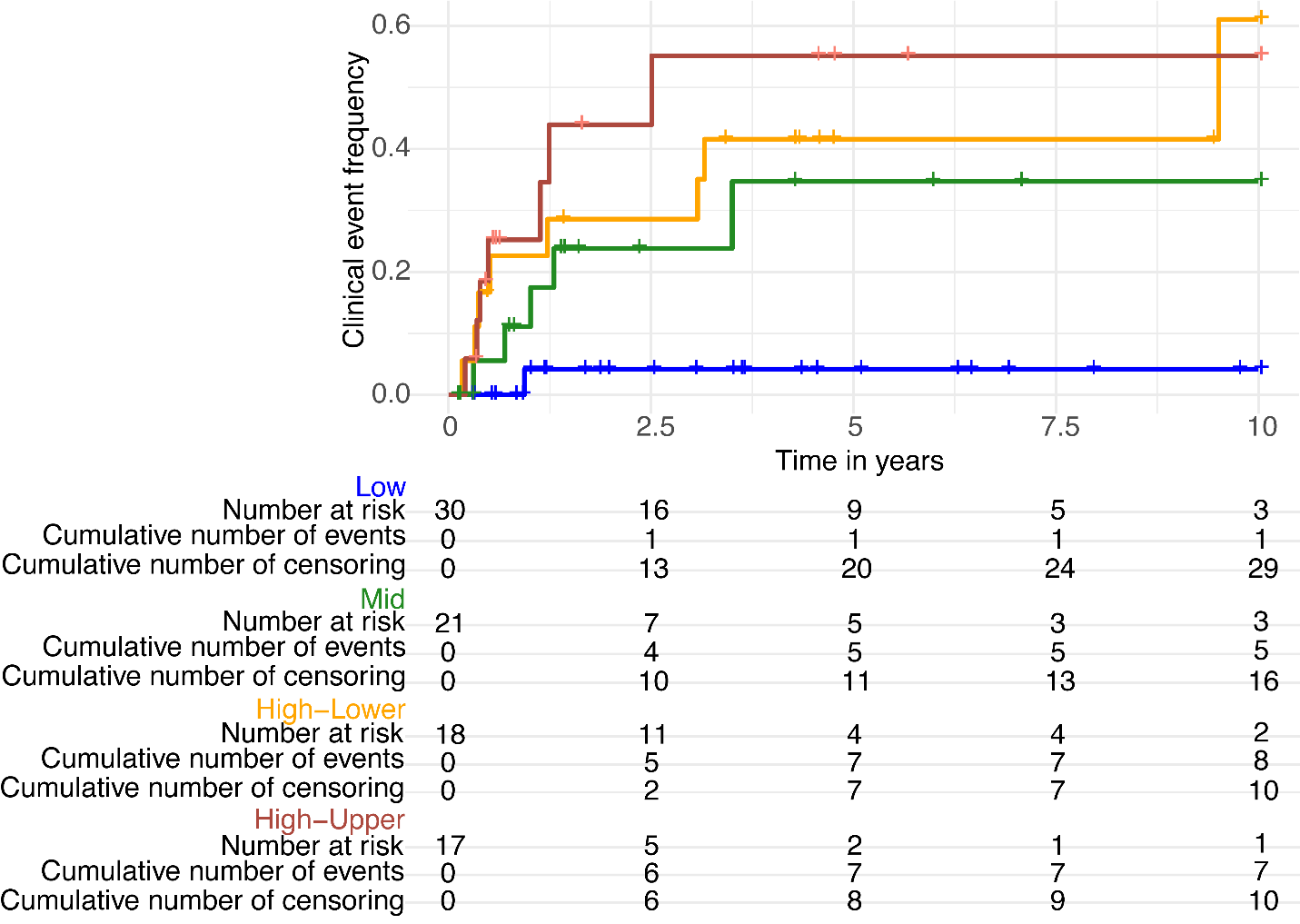


**Supplementary Figure 3: Time to conversion to MS by IgG index–based stratified subgroups.** Kaplan-Meier curves illustrating cumulative conversion rates over a 10-year interval stratified by IgG index tertile-derived subgroups: Low (≤ 0.52) (blue, N = 30), Mid (>0.52–0.70) (green, N = 21), High-Lower (>0.70–0.97) (orange, N = 18), and High-Upper (>0.97–1.61) (brown, N = 17). Individuals in the High-Upper and High-Lower subgroups showed elevated risks of MS conversion compared to the Low group. The global log-rank test indicated significant differences across subgroups (P = 0.0053), and Bonferroni-adjusted pairwise comparisons confirmed differences for High-Lower (P = 0.045) and High-Upper (P = 0.0075) versus Low. The observed divergence in survival curves occurred primarily within the first five years, suggesting a dose-dependent effect of IgG index values on early MS conversion risk.

**Reference**

1. Lebrun-Frenay C, Okuda DT, Siva A, et al. The radiologically isolated syndrome: revised diagnostic criteria. *Brain*. Aug 1 2023;146(8):3431-3443. doi:10.1093/brain/awad073
